# Effects of grazing exclusion on soil properties, fungal community structure, and diversity in different grassland types

**DOI:** 10.1002/ece3.11056

**Published:** 2024-03-01

**Authors:** Shijie Zhou, Yiqiang Dong, Helong Yang, Suwen Yang, Asitaiken Julihaiti, Zeyu Liu, Tingting Nie, Anjing Jiang, Yue Wu, Shazhou An

**Affiliations:** ^1^ School of Grassland Xinjiang Agricultural University Urumqi China; ^2^ Key Laboratory of Grassland Resources and Ecology Autonomous Region Urumqi Xinjiang China; ^3^ Key Laboratory of Grassland Resources and Ecology Ministry of Education Urumqi China

**Keywords:** diversity, driving factor, enclosure, fungi, soil physical and chemical properties

## Abstract

Soil fungi are involved in the decomposition of organic matter, and they alter soil structure and physicochemical properties and drive the material cycle and energy flow in terrestrial ecosystems. Fungal community assembly processes were dissimilar in different soil layers and significantly affected soil microbial community function and plant growth. Grazing exclusion is one of the most common measures used to restore degraded grasslands worldwide. However, changes in soil fungal community characteristics during grazing exclusion in different types of grasslands are unknown. Here, we investigated the effects of a 9‐year grazing exclusion on soil properties, fungal community composition, and diversity in three grassland types (temperate desert, temperate steppe, and mountain meadow). The results showed that (1) in the 0–5 cm soil layer, grazing exclusion significantly increased the differences in SWC, SOC, KN, and N:P among the three grassland types, while the final pH, BD, TP, C:N, and C:P values were consistent with the results before exclusion. In the 5–10 cm soil layer, grazing exclusion significantly increased total phosphorus (TP) in temperate deserts by 34.1%, while significantly decreasing bulk density (BD) by 9.8% and the nitrogen: phosphorus ratio (N:P) by 47.1%. (2) The soil fungal community composition differed among the grassland types, For example, significant differences were found among the three grassland types for the *Glomeromycota* and *Mucoromycota.* (3) Under the influence of both grazing exclusion and grassland type, there was no significant change in soil fungal alpha diversity, but there were significant differences in fungal beta diversity. (4) Grassland type was the most important factor influencing changes in fungal community diversity, and vegetation cover and soil kjeldahl nitrogen were the main factors influencing fungal diversity. Our research provides a long‐term perspective for better understanding and managing different grasslands, as well as a better scientific basis for future research on grass–soil–microbe interactions.

## Graphical abstract

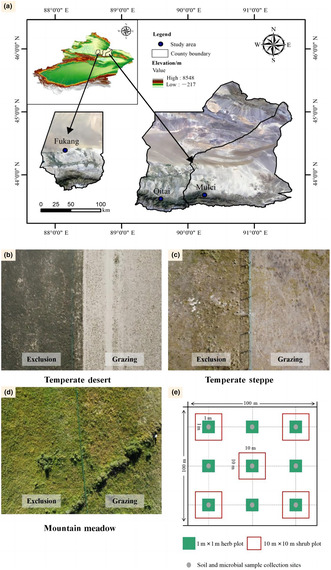

## 1 INTRODUCTION

Grasslands cover 20% of the land surface and play an important role in preventing soil erosion and supporting livestock farming in semi‐arid areas (Jing et al., 2013, 2014). In recent decades, grassland degradation has become widespread globally due to increased human activity and climate change (Jalaludin et al., 2020; O'Mara, 2012). China has approximately 400 million hectares of grasslands of various types, accounting for approximately 41.7% of the country's land area (Xu et al., 2016), and more than 90% of the grasslands are in a degraded state (Wang et al., 2020). The low quality of grasslands leads to a severe weakening of grassland ecological functions, the degradation of soil nutrients (Jing et al., 2014), and disruption in the original balance of the system, transforming them into a fragile ecosystems (Zheng & Zhu, 2012). How to effectively rehabilitate degraded grasslands is an enormous scientific and technical challenge (Wang et al., 2020). The most widely used measure for the ecological management of degraded grasslands is grazing exclusion, which can restore vegetation biomass (Su et al., 2005), improve soil fertility (Mekuria et al., 2007), and increase grassland use efficiency (Yang et al., 2005). Previous studies have focused on the effects of grazing exclusion on plant communities and soil properties (Dong et al., 2016), while research on the effects on soil fungal communities has been limited.

Soil fungal communities are sensitive to environmental change. Changes in local fungal communities are recognized as an early warning of problems and an important indication of changes in soil ecosystems (Kennedy & Smith, 1995; Somova & Pechurkin, 2001). Soil fungi also play a key role in regulating material cycling in terrestrial ecosystems (Bais et al., 2006). The structure and function of soil fungal communities in degraded grasslands are often limited (Zhou et al., 2011). Soil fungi can decompose macromolecules such as lignin and cellulose in plant residues, and play important roles in soil nutrient accumulation, transformation, and cycling (Grau et al., 2017). Fungal community assembly processes were dissimilar in different soil layers and significantly affected soil microbial community function and plant growth. In desert and semi‐arid regions, soil thickness had an important effect on vegetation and soil microbial communities changed significantly with soil thickness. Fungal community assembly was dominated by stochastic processes in both the 0–5 cm soil layer and the 5–10 cm soil layer of both temperate desert grasslands and temperate steppes after fencing (Fierer et al., 2003; Khumalo et al., 2008). Therefore, it is necessary to assess changes in soil fungal communities under grazing exclusion conditions for soil and vegetation restoration and to predict changes in grassland ecosystem dynamics under conditions of environmental change.

Different types of grasslands have integral roles in maintaining the stability and diversity of grassland ecosystems (Yang, Sun, et al., 2018; Yang, Zhang, et al., 2018). Numerous studies have shown that the response of soil bacteria and fungi to grazing exclusion is not consistent across different grassland types. Soil microbial richness indices in alpine grasslands under grazing exclusion were higher than those in grazed grasslands of the same type, and grazing changed the structure of soil microbial communities (Jing et al., 2021). In alpine meadows, the total number of soil microorganisms was significantly lower after grazing exclusion (Xie et al., 2017). Sun et al. (2018) found that grazing exclusion had no significant effect on the overall numbers of soil bacteria and fungi in a *Seriphidium transiliense* desert. Therefore, the state of knowledge regarding the variation in grazing exclusion of soil bacteria and fungi in different grassland types is insufficient, and further research is necessary. Fungi can be closely associated with host plants through symbiosis or parasitism (García‐Guzmán & Heil, 2014; Maron et al., 2011; Zhu et al., 2017) and play a dominant role in soil nutrient cycling (Mcguire et al., 2010; Treseder & Holden, 2013). However, studies indicate that grazing exclusion does not significantly affect the diversity of fungal communities, probably due to the greater stability of fungi than that of bacteria (Cheng et al., 2016; Zhang et al., 2018). The results of studies on the effects of grazing exclusion on the characteristics of soil fungal communities in different grassland types are still unclear, which limits our knowledge of the changes in soil fungal communities after grazing exclusion.

The total area of natural grassland in Xinjiang is approximately 5.7 × 10^7^ ha, accounting for 34.4% of the land area (Xu, 1993), and this area is a valuable resource for the development of the livestock industry and national economy in Xinjiang. Among the Xinjiang grasslands, temperate desert, temperate steppe, and mountain meadow grasslands account for 48.28% of the total grassland area (Wang et al., 2006). Considering the importance of grasslands in Xinjiang, it is vital to understand the effects of grazing exclusion on soil fungal communities in different grassland types in the region. This knowledge will have great significance for grassland managers and policymakers. Here, we conducted a regional field experiment to assess the effects of grazing exclusion, grassland type, and their interaction on soil physicochemical properties, and soil fungal community composition and diversity in combination with plant community characteristics and soil properties. We hypothesized that (1) grazing exclusion and grassland type would alter fungal communities and diversity. (2) Fungal composition and diversity respond differently to grazing exclusion across grassland types. (3) Vegetation characteristics and soil nutrient content are the main drivers of changes in soil fungal diversity under grazing exclusion and grassland‐type treatments. Our main objectives were to (1) clarify the effects of grazing exclusion and grassland type and their interactions on fungal communities and diversity; and (2) investigate the main drivers of soil fungal diversity under grazing exclusion and grassland‐type treatments. This study provides a long‐term perspective for a better understanding and management of different grasslands and a better scientific basis for future research on grass–soil–microbe interactions.

## 2 MATERIALS AND METHODS

### 2.1 Study area

**FIGURE 1:**
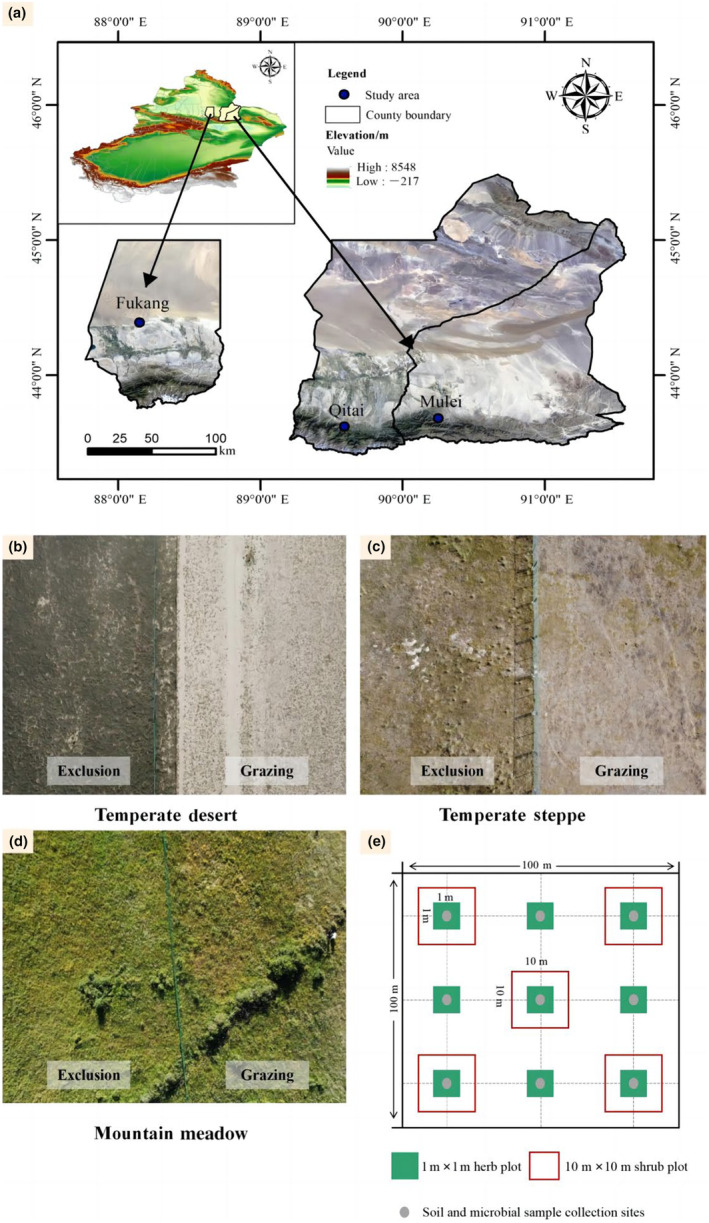
Location of sampling points (a). Each grassland type includes a paired no‐grazing and grazing site; (b) a temperate desert sample site; (c) a temperate grassland sample site; (d) a mountain meadow sample site; and (e) the sample layout.

The study area is located in Changji Hui Autonomous Prefecture (43.62°–44.39° N, 88.15°–90.25° E, 514–2611 m elevation) in the eastern section of the northern slope of the Tianshan Mountains in Xinjiang (Figure 1). The region has a typical temperate arid climate, with high temperatures and rainy summers and cold, dry winters, with an average annual precipitation of 150–300 mm and an average annual temperature of 5–6°C (Zhou et al., 2012). In the temperate desert zone (Fukang city), the main dominant plants are *Haloxylon ammodendron* (C.A. Mey) Bunge and *Seriphidium santolinum (Schrenk) Poljakov*; in the temperate steppe zone (Mulei County), the main dominant plant species are *Festuca ovina* and *Carex liparocarpos*; and in the mountain meadow zone (Qitai County), the dominant plants are mostly grasses and weedy grasses (Astiken et al., 2023).

### 2.2 Experimental design and field sampling

In July 2021, three areas (Fukang City, Mubi County, and Qitai County) on the eastern section of the northern slope of Tianshan were selected. Each area corresponded to one grassland type: temperate desert, temperate steppe, and mountain meadow. The test sites were all located within the national fixed monitoring sites and have been fenced since 2012. The fenced areas are surrounded by spring and autumn grazing areas for sheep, and the grazing intensity is moderate (0.6–1.0 sheep/ha). At each site, paired plots (a long‐term grazing plot and a grazing exclusion plot) were sampled. All paired plots shared soil type and geographical conditions, including slope, elevation, and topography. Three sample lines were set up at 50 m intervals in each of the grazing exclusion and grazing areas, and three 1 m × 1 m herb samples were laid out at approximately 50 m intervals on each sample line (a total of 54 small samples). If there were shrubs in the plot, five additional shrub plots (10 m × 10 m) were measured. The plant species present in each square were recorded. For each plant species, its cover, average height, density, and biomass were documented. Plant coverage was measured using the projection method, natural heights were measured using a ruler, and individual quantities (densities) of each species were recorded with the help of statistical methods. The aboveground biomass of each species was estimated in each sampling plot by clipping the plant from the soil surface using scissors. The collected plant material was brought back to the laboratory for treatment (dried at 80°C for 24 h to a constant weight). All the plant data are presented in Table S1. Soil samples were collected at two depths: 0–5 and 5–10 cm. The samples from the same sample line and depth were mixed and placed in sealed bags. The samples were taken back to the laboratory in a vehicle refrigerator (−20°C). Half of the samples were stored at 4°C, while the rest were air‐dried at room temperature. Plant roots, gravel, and other debris were removed from the air‐dried samples. The remaining soil was ground, mixed, and sieved through 1 and 0.25 mm sieves for analysis.

### 2.3 Plant community diversity measurement

According to Whittaker's scientific and comprehensive discussion on an evaluation index of plant diversity, the α‐diversity index is often used to study the species diversity within a community caused by the differentiation of interspecific niches within a community and is a commonly used evaluation index in ecological research (Whittaker, 1972). This study used the method proposed by Whittaker. The community structure attributes were characterized by the importance value (IV), Patrick richness index (*R*), Simpson dominance index (*D*), Shannon–Wiener diversity index, and Pielou index (*E*) (Wu et al., 2009), and they were calculated as follows:

(1) $$\text{Importance value}\;\left(\mathrm{IV}\right):{\mathrm{IV}}_{i}=\left(H_{\mathrm{ri}}+C_{\mathrm{ri}}+D_{\mathrm{ri}}+B_{\mathrm{ri}}\right)/4$$

(2) $$\text{Patrick richness index}\;\left(R\right):R=S$$

(3) $$\text{Simpson dominance index}\;\left(D\right):D=1-\sum {\mathrm{IV}}_{i}^{2}$$

(4) $$\text{Shannon}–\text{Wiener diversity index}\;\left(H\right):H=-\sum {\mathrm{IV}}_{i}{\text{InIV}}_{i}$$

(5) $$\text{Pielou evenness index}\;\left(E\right):E=\left(-\sum {\mathrm{IV}}_{i}\ln{\mathrm{IV}}_{i}\right)/\ln S$$

where *H*_ri_ represents the relative height, *C*_ri_ indicates the relative coverage, *D*_ri_ represents the relative density, *B*_ri_ is the relative biomass, *S* represents the number of species in the community, and IV_*i*_ is a representation of the importance value of species *i*.

### 2.4 Soil properties analysis

The pH value was determined by the acidometer method (water–soil mass ratio of 2.5:1; PHS‐3G digital pH meter, Shanghai) (Cao et al., 2017). The soil water content (SWC) was determined gravimetrically by drying the soil samples (105°C, 24 h). The bulk density (BD) was measured gravimetrically after oven drying (105°C, 24 h). The soil organic carbon (SOC) was determined using the dichromate oxidation method (Walkley & Black, 1934). Total phosphorus (TP) was measured using the Mo‐Sb colorimetric method with a spectrophotometer (Lambda25 UV‐vis Spectrometer, United States). Kjeldahl nitrogen (KN) was determined using the Kjeldahl method.

### 2.5 DNA extraction and Illumina NovaSeq sequencing

Total genomic DNA from samples was extracted using the CTAB method (Murray & Thompson, 1980). DNA concentration and purity were monitored on 1% agarose gels. According to the concentration, DNA was diluted to 1 ng/μL using sterile water.

Depending on the position of the rDNA of the measured sample, which lies between 18 s and 5.8 s, ITS rRNA genes of distinct regions (ITS1‐1F) were amplified using specific primers ITS1‐1F‐F (5′‐CTTGGTCATTTAGAGGAAGTAA‐3′) and ITS1‐1F‐R (5′‐GCTGCGTTCTTCATCGATGC‐3′) with the barcodes (Bokulich & Mills, 2013; Hugerth et al., 2014). All PCR reactions were carried out with 15 μL of Phusion® High‐Fidelity PCR Master Mix (New England Biolabs), 2 μM of forward and reverse primers, and approximately 10 ng of template DNA. Thermal cycling consisted of initial denaturation at 98°C for 1 min, followed by 30 cycles of denaturation at 98°C for 10 s, annealing at 50°C for 30 s, elongation at 72°C for 30 s, and finally, 72°C for 5 min. The same volume of 1× TAE buffer was mixed with the PCR products, and electrophoresis was performed on a 2% agarose gel for detection. PCR products were mixed in equidensity ratios. Then, the mixture of PCR products was purified with a Qiagen Gel Extraction Kit (Qiagen, Germany).

Sequencing libraries were generated using a TruSeq® DNA PCR‐Free Sample Preparation Kit (Illumina, USA) following the manufacturer's recommendations, and index codes were added. The library quality was assessed on a Qubit@ 2.0 Fluorometer (Thermo Scientific). Finally, the library was sequenced on an Illumina NovaSeq platform, and 250 bp paired‐end reads were generated.

### 2.6 Statistics and analysis

All data are shown as the mean and standard error. Diversity metrics were calculated using the core‐diversity plugin within QIIME2. Feature‐level α‐diversity indices, such as the Simpson index, Chao1 richness index, and Shannon diversity index, were calculated to estimate the fungal diversity. Data on soil physicochemical properties and microbial diversity indices were analyzed by independent *T*‐test, one‐way ANOVA, multifactor ANOVA, and Pearson's correlation analysis using SPSS 26.0. Bar graphs were generated in OriginPro 2021 (Originlab Corporation, USA). Principal coordinate analysis (PCoA) using version R4.2.1 and Bray–Curtis distances were used to visualize the effects of grazing exclusion and grassland type on fungal β‐diversity. PERMANOVA (permutational multivariate analysis of variance) was performed in the ‘vegan’ package of R using the ANOSIM function to test for the significance of differences in community and plotted using the “ggplot2” package in R 4.2.1. Finally, we implemented structural equation modeling (SEM) based on the lavaan package (Rosseel, 2011) to assess the effects of grazing exclusion and grassland type on fungal diversity through changes in plant and soil abiotic variables; the statistical analyses of which were carried out using R version 4.2.1.

## 3 RESULTS

### 3.1 Soil physicochemical properties as affected by grazing exclusion and grassland type

In the 0–5 cm soil layer (Table 1), grazing exclusion significantly affected the physicochemical parameters of all three studied grassland types. In the temperate desert, grazing exclusion resulted in a 36.36% significant increase in TP. In the temperate steppe, SWC and SOC increased significantly, while BD was reduced significantly by 16.67%. In the mountain meadow, KN significantly decreased. Grazing exclusion significantly increased the differences in SWC, SOC, KN, and N:P among the three grassland types, while the final pH, BD, TP, C:N, and C:P values were consistent with the results before exclusion (Table S2). In the 5–10 cm soil layer, grazing exclusion significantly increased TP in temperate deserts by 34.1% while significantly decreasing BD by 9.8% and N:P by 47.1%. In temperate steppes, C:N was significantly reduced. In mountain meadows, grazing exclusion resulted in a significant increase in SWC and SOC and significant decreases in BD. After grazing exclusion, C:N was significantly higher in temperate deserts than in temperate steppe and mountain meadows, while SOC and BD were not significantly different between temperate steppe and mountain meadows (Table S3).

**TABLE 1:** Soil physicochemical properties as affected by grazing exclusion and grassland type.

Index	Treatment	Temperate desert		Temperate steppe		Mountain meadow	
		0–5 cm	5–10 cm	0–5 cm	5–10 cm	0–5 cm	5–10 cm
pH	Grazing	9.59 ± 0.12Aa	9.62 ± 0.17Aa	6.66 ± 0.09Ab	6.73 ± 0.07Ab	6.78 ± 0.10Ab	6.67 ± 0.16Ab
	Exclusion	9.39 ± 0.23Aa	9.67 ± 0.21Aa	6.66 ± 0.04Ab	6.64 ± 0.04Ab	6.98 ± 0.07Ab	6.52 ± 0.11Ab
SWC (%)	Grazing	2.00 ± 1.00Ab	2.00 ± 0.00Ab	17.00 ± 2.00Ba	19.00 ± 2.00Aa	18.00 ± 2.00Aa	17.00 ± 1.00Ba
	Exclusion	2.00 ± 0.00Ac	2.00 ± 0.00Ab	23.00 ± 0.00Aa	18.00 ± 1.00Aa	18.00 ± 2.00Ab	21.00 ± 1.00Aa
BD (g/cm^3^)	Grazing	1.48 ± 0.07Aa	1.53 ± 0.02Aa	0.78 ± 0.02Ab	0.78 ± 0.02Ac	0.82 ± 0.05Ab	1.02 ± 0.05Ab
	Exclusion	1.37 ± 0.06Aa	1.38 ± 0.04Ba	0.65 ± 0.01Bb	0.78 ± 0.01Ab	0.74 ± 0.02Ab	0.84 ± 0.02Bb
SOC (g/kg)	Grazing	1.22 ± 0.15Ab	0.87 ± 0.17Ac	98.44 ± 1.47Ba	88.05 ± 8.33Aa	84.92 ± 12.60Aa	60.77 ± 0.39Bb
	Exclusion	2.92 ± 0.62Ac	1.27 ± 0.08Ab	119.88 ± 6.87Aa	70.57 ± 3.98Aa	81.60 ± 10.36Ab	73.26 ± 2.16Aa
KN (g/kg)	Grazing	0.13 ± 0.06Ab	0.07 ± 0.00Ab	8.25 ± 1.18Aa	8.75 ± 0.73Aa	10.00 ± 0.19Aa	5.60 ± 1.30Aa
	Exclusion	0.32 ± 0.08Ac	0.05 ± 0.01Ab	10.89 ± 0.39Aa	7.60 ± 0.31Aa	8.41 ± 0.10Bb	7.95 ± 0.39Aa
TP (g/kg)	Grazing	0.44 ± 0.01Bb	0.44 ± 0.01Bb	0.85 ± 0.09Aa	0.95 ± 0.04Aa	0.99 ± 0.02Aa	0.68 ± 0.11Aab
	Exclusion	0.60 ± 0.03Ab	0.59 ± 0.02Ab	0.98 ± 0.03Aa	0.86 ± 0.02Aa	0.93 ± 0.05Aa	0.91 ± 0.03Aa
C:N	Grazing	14.40 ± 6.32Aa	11.78 ± 2.12Aa	12.50 ± 2.01Aa	10.05 ± 0.21Aa	8.45 ± 1.12Aa	12.58 ± 3.70Aa
	Exclusion	9.34 ± 0.89Aa	24.80 ± 4.53Aa	11.00 ± 0.44Aa	9.28 ± 0.15Bb	9.72 ± 1.34Aa	9.29 ± 0.76Ab
C:P	Grazing	2.81 ± 0.40Ab	2.00 ± 0.37Ab	118.06 ± 13.26Aa	92.34 ± 5.84Aa	86.00 ± 11.92Aa	95.39 ± 29.55Aa
	Exclusion	4.80 ± 0.83Ab	2.16 ± 0.17Ab	122.35 ± 6.85Aa	81.57 ± 3.35Aa	88.63 ± 11.99Aa	80.56 ± 7.85Aa
N:P	Grazing	0.30 ± 0.14Ab	0.17 ± 0.01Ab	9.58 ± 0.41Ba	9.17 ± 0.44Aa	10.16 ± 0.12Aa	8.05 ± 0.81Aa
	Exclusion	0.53 ± 0.11Ac	0.09 ± 0.02Bb	11.11 ± 0.20Aa	8.79 ± 0.23Aa	9.15 ± 0.48Ab	8.71 ± 0.22Aa

*Note*: The means (±SE) for each variable followed by different uppercase letters indicate significant differences between grazing and exclusion sites (*p* < .05). The lowercase letters indicate significant differences among different grassland types (*p* < .05). Based on two‐way variance comparisons.

Abbreviations: BD, bulk density; C:N, carbon:nitrogen; C:P, carbon:phosphorus; KN, Kjeldahl nitrogen; N:P, nitrogen:phosphorus; SOC, soil organic carbon; SWC, soil water content; TP, total phosphorus.

### 3.2 Soil fungal community OTU numbers as affected by grazing exclusion and grassland type

**FIGURE 2:**
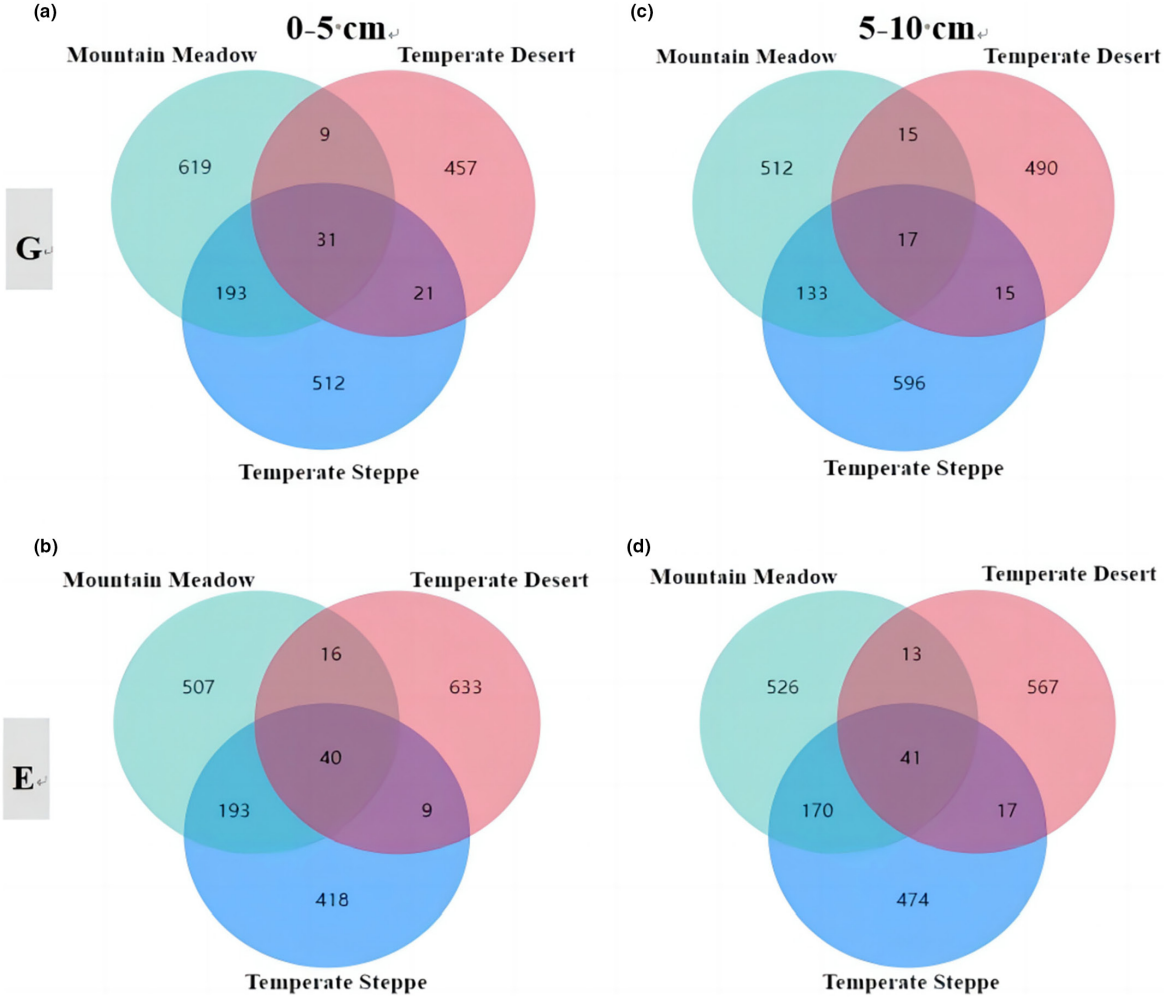
Venn diagram of fungal communities in different grassland types based on OTU abundance. Different colors represent grassland types. The letter G denotes grazing, and the letter E denotes grazing exclusion. Figure (a) shows the abundance of soil OTU in the 0–5 cm soil layer under grazing; Figure (b) shows the abundance of soil OTU in the 5–10 cm soil layer under grazing; Figure (c) shows the abundance of soil OTU in the 0–5 cm soil layer under grazing exclusion; Figure (d) shows the abundance of soil OTU in the 5–10 cm soil layer under grazing exclusion.

Wayne analysis (Figure 2) showed that in the 0–5 cm soil layer (Figure 2a), the number of fungal operational taxonomic units (OTUs) in mountain meadows (619) accounted for 38.98% of the total OTUs; the number of fungal OTUs in temperate steppe (512) accounted for 32.24% of the total OTUs; and the number of fungal OTUs in temperate deserts (457) accounted for 28.78% of the total OTUs. After exclusion (Figure 2b), the number of fungal OTUs in mountain meadows and temperate steppes decreased by 18.1% and 18.36%, respectively, compared with those before exclusion, but the number of fungal OTUs in temperate deserts increased by 38.51% compared with those before exclusion. In the 5–10 cm soil layer (Figure 2c), the number of fungal OTUs in mountain meadows (512) accounted for 32.04% of the total OTUs; the number of fungal OTUs in temperate steppes (596) accounted for 37.30% of the total OTUs; and the number of fungal OTUs in temperate deserts (490) accounted for 30.66% of the total OTUs. After exclusion (Figure 2d), the number of fungal OTUs in temperate steppes decreased by 20.47% compared with that before exclusion, while the number of fungal OTUs in mountain meadows and temperate deserts increased by 2.7% and 15.71%, respectively, compared with that before exclusion. In addition, we illustrate the OTUs situation between grazing and grazing exclusion for different grassland types (Figure S1).

### 3.3 Soil fungal community composition as affected by grazing exclusion and grassland type

**FIGURE 3:**
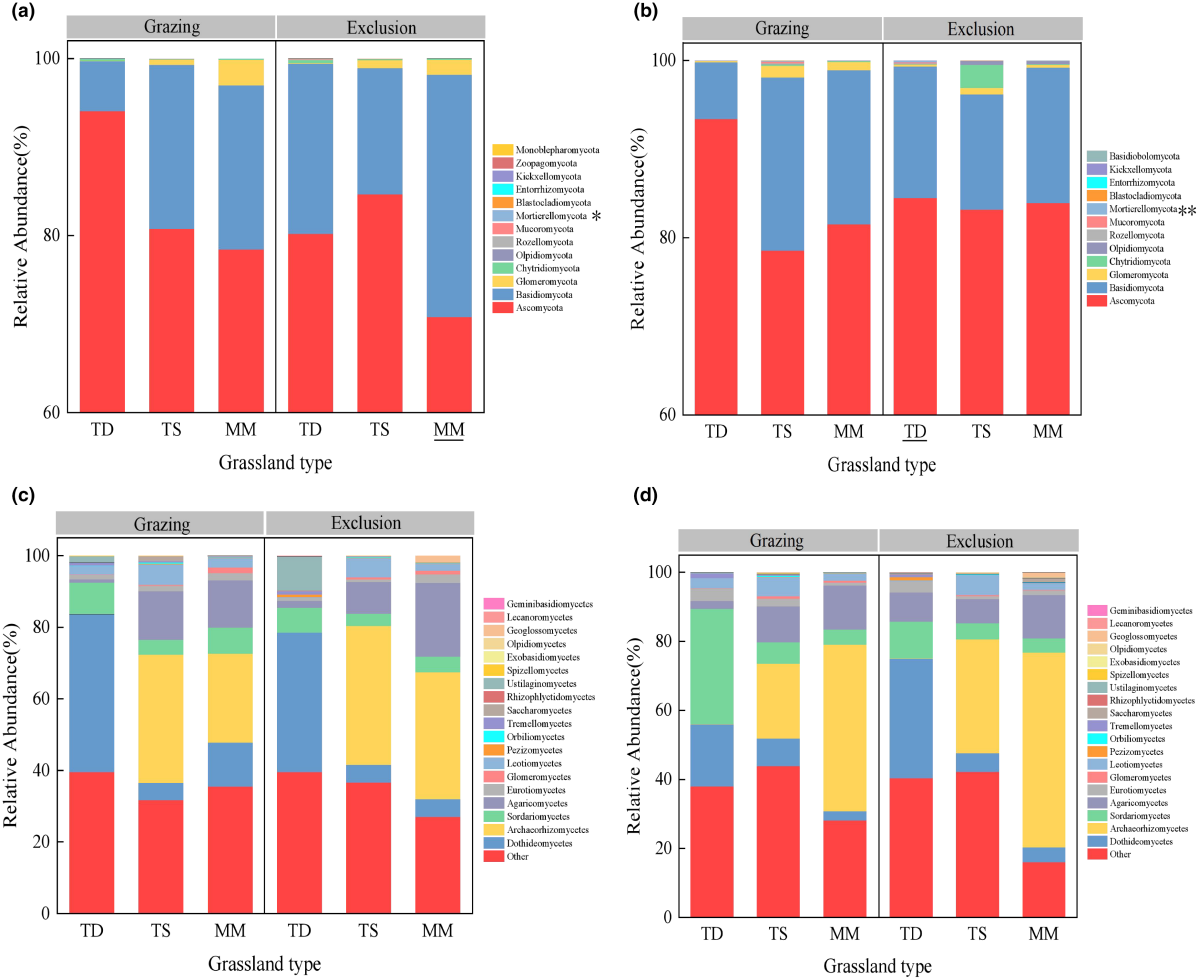
Effect of grazing exclusion and grassland type on soil fungi phylum and class community composition. (a) The composition of the soil fungal phylum in the 0–5 cm soil layer; (b) the composition of the soil fungal phylum in the 5–10 cm soil layer; (c) the composition of the soil fungal class in the 0–5 cm soil layer; and (d) the composition of the soil fungal class in the 5–10 cm soil layer. MM, mountain meadow; TD, temperate desert; TS, temperate steppe. Where *represents significant and **represents highly significant.

At the phylum level (Figure 3a), Ascomycota and Basidiomycota were the dominant fungi in the three grassland types in the 0–5 cm soil layer before exclusion. After exclusion, Ascomycota decreased by 14.72% and 9.74% in temperate deserts and mountain meadows, respectively, and increased by 4.82% in temperate steppes. Basidiomycota increased by 238.8% and 47.68% in temperate deserts and mountain meadows, respectively, but decreased by 23.12% in temperate steppes. Significant differences were found between the three grassland types for the Glomeromycota and Mucoromycota (*p* < .05; Table S4). In the 5–10 cm soil layer (Figure 3b), the main fungi of the three grassland types were the same as those in the 0–5 cm soil layer. It is worth mentioning that the changes in *Mortierellomycota* were we found to be significantly lower after grazing exclusion in the 0–5 soil layer in mountain meadows (*p* < .05) and significantly higher in the 5–10 soil layer in temperate deserts (*p* < .001). In addition, grassland type significantly influenced the Blastocladiomycota (*p* < .05; Table S5). At the class level in the 0–5 cm soil layer (Figure 3c), regardless of grazing or grazing exclusion, Dothideomycetes, Archaeorhizomycetes, Sordariomycetes, and Agaricomycetes were dominant in three grassland types studied, while in the temperate desert, Archaeorhizomycetes almost disappeared. This result illustrates the different responses of fungi to environmental changes. In the 5–10 cm soil layer (Figure 3d), *Sordariomycetes* increased significantly by 279.32% in the temperate desert before exclusion compared to the 0–5 cm soil layer. Grassland type had a highly significant effect on *Ustilaginomycetes* (*p* < .001) and a significant effect on *Eurotiomycetes* and *Orbiliomycetes* (*p* < .05). In addition, grassland type had a highly significant effect on *Dothideomycetes* and *Archaeorhizomycetes* in both the 0–5 cm and the 5–10 cm soil layers (*p* < .001) (Tables S6 and S7).

### 3.4 Soil fungal community diversity as affected by grazing exclusion and grassland type

**FIGURE 4:**
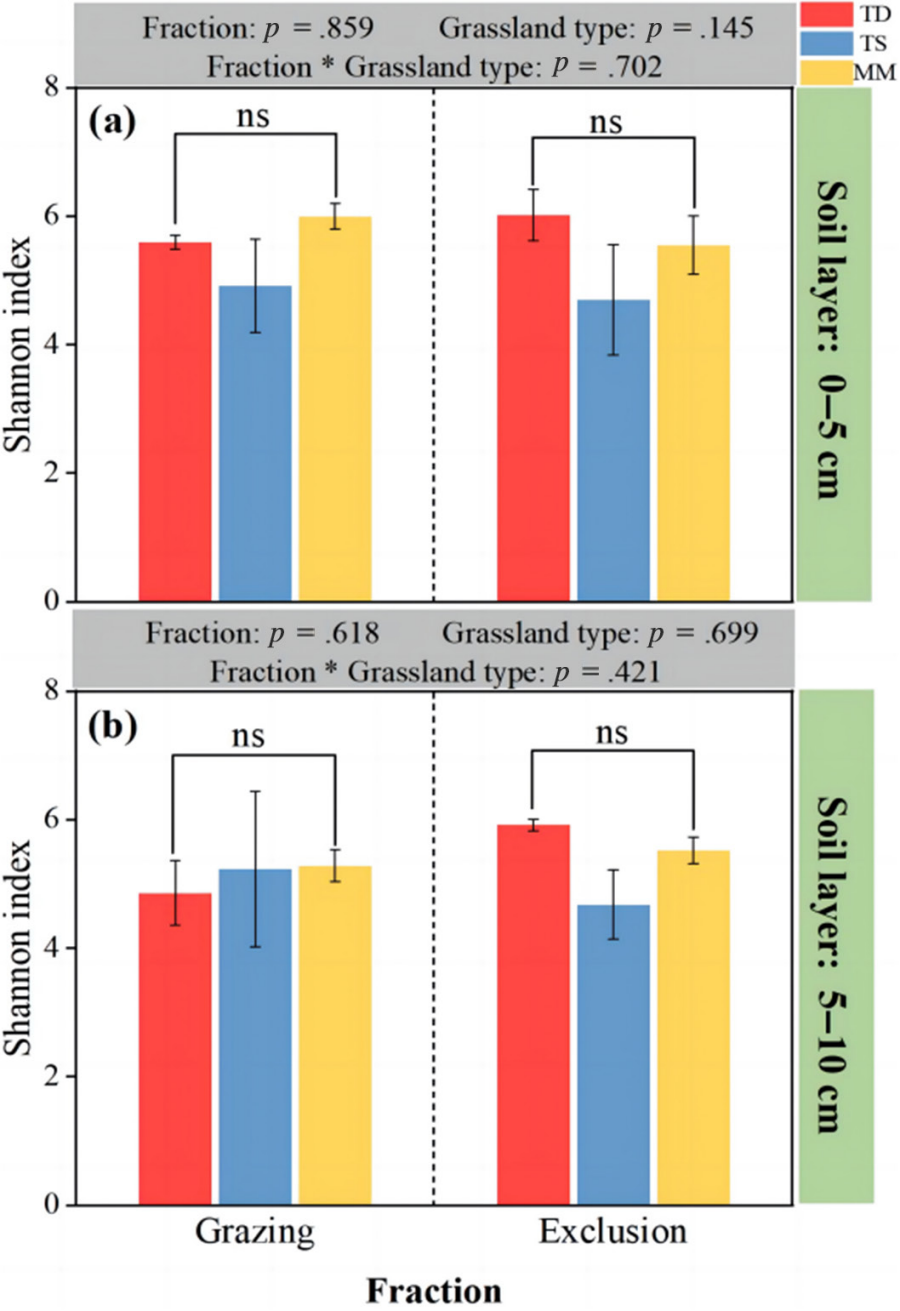
Soil fungal α‐diversity as affected by grazing exclusion and grassland type. Error bars indicate standard errors (three replicate sites). The lowercase letters indicate significant differences among different grassland types (*p* < .05). MM, mountain meadow; TD, temperate desert; TS, temperate steppe. (a) Represents the 0–5 cm soil fungal Shannon index; (b) represents the 5–10 cm soil fungal Shannon index.

Soil fungal α‐diversity was not significantly altered by grazing exclusion, grassland type, or the interaction between the two in either the 0–5 cm or the 5–10 cm soil layers (*p* > .05; Figure 4).

**FIGURE 5:**
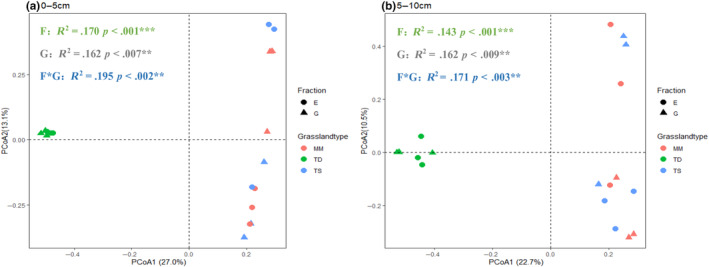
Fungal community structure assessed by β‐diversity patterns using the principal coordinate analysis plots of Bray–Curtis distances. Different shapes represent grazing exclusion or grazing soils, and colors represent grassland types. E, exclusion; G, grazing; MM, mountain meadow; TD, temperate desert; TS, temperate steppe. ANOSIM similarity analysis was used to test the significance between groups.

A principal coordinate analysis (PCoA) of soil fungal communities based on Bray–Curtis distances showed that PCoA1 and PCoA2 explained 27.0% and 13.1% of the variation in soil fungal communities in the 0–5 cm soil layer, respectively, with a cumulative contribution of 40.1% (Figure 5a). ANOSIM analysis showed significant differences among soil fungal communities regardless of grazing exclusion or grassland type and the interaction of the two (*p* < .001; *p* < .007; *p* < .002). In the 5–10 cm soil layer, PCoA1 and PCoA2 explained 22.7% and 10.5% of the variation in soil fungal communities, respectively, with a cumulative contribution of 33.2% (Figure 5b). ANOSIM analysis showed significant differences among soil fungal communities regardless of grazing exclusion or grassland type and the interaction of the two (*p* < .001; *p* < .009; *p* < .003).

### 3.5 Correlations of grazing exclusion and grassland types with plant, soil, and fungal composition and diversity

**FIGURE 6:**
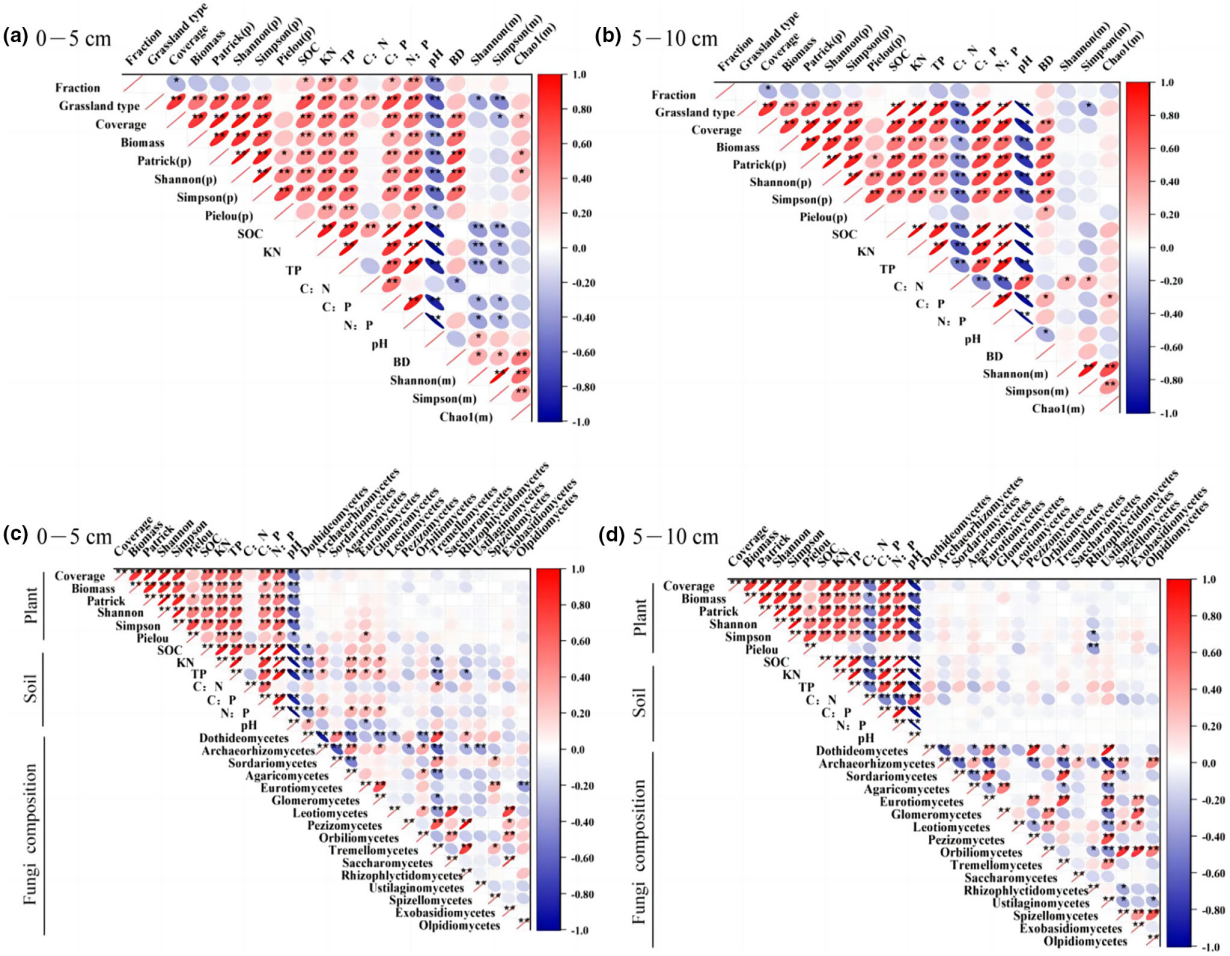
Correlation of grazing exclusion and grassland types with plant, soil, and fungal composition and diversity. Fraction represents grazing exclusion and grazing; grassland type represents temperate desert, temperate steppe, and mountain meadow. Plant (p); microorganism (m). Circle size and color represent the Pearson correlation coefficient. Figure (a) shows the correlation analysis of grazing exclusion and grassland types with plant, soil and diversity in the 0–5 cm soil layer; Figure (b) shows the correlation analysis of grazing exclusion and grassland types with plant, soil and diversity in the 5–10 cm soil layer; Figure (c) shows the correlation analysis of grazing exclusion and grassland types with plant, soil and fungal composition in the 0–5 cm soil layer; Figure (d) shows the correlation analysis of grazing exclusion and grassland types with plant, soil and fungal composition in the 5–10 cm soil layer.

In the 0–5 cm soil layer (Figure 6a,c), all soil indices except the C:N soil index were significantly correlated with the grazing exclusion treatment; additionally, plant coverage was significantly correlated with the no‐grazing treatment, but fungal α‐diversity was not significantly correlated with the no‐grazing treatment. Except for the plant Pielou index and fungal Chao1 index, all other plant, soil, and fungal diversity indicators were significantly or highly significantly correlated with grassland type. In addition, we found that soil SOC, KN, TP, C:P, N:P, and BD were significantly or highly significantly negatively correlated with the fungal Shannon index and Simpson index. In terms of fungal composition, *Eurotiomycetes* was significantly and positively correlated with the plant Pielou index, KN, TP, N:P, and pH, and *Dothideomycetes* was significantly and negatively correlated with the SOC but significantly and positively correlated with pH. We found that soil KN, TP, and N:P were significantly or highly significantly correlated with *Dothideomycetes*, *Archaeorhizomycetes*, *Agaricomycetes*, and *Glomeromycetes*.

In the 5–10 cm soil layer (Figure 6b,d), plant cover was significantly correlated with grazing closure treatments, and all plant, soil, and fungal diversity indices, except for the plant Pielou index, fungal Shannon index, fungal Chao1 index, and BD, were significantly or highly significantly correlated with grassland type. In addition, we found that soil C:N was significantly and positively correlated with the fungal Shannon index and Simpson index, and soil C:P was significantly and positively correlated with the fungal Chao1 index. In terms of fungal composition, only *Rhizophlyctidomycetes* was significantly negatively correlated with the plant Simpson's index and highly significantly negatively correlated with the plant Pielou's index.

### 3.6 Potential drivers of soil fungal community diversity

**FIGURE 7:**
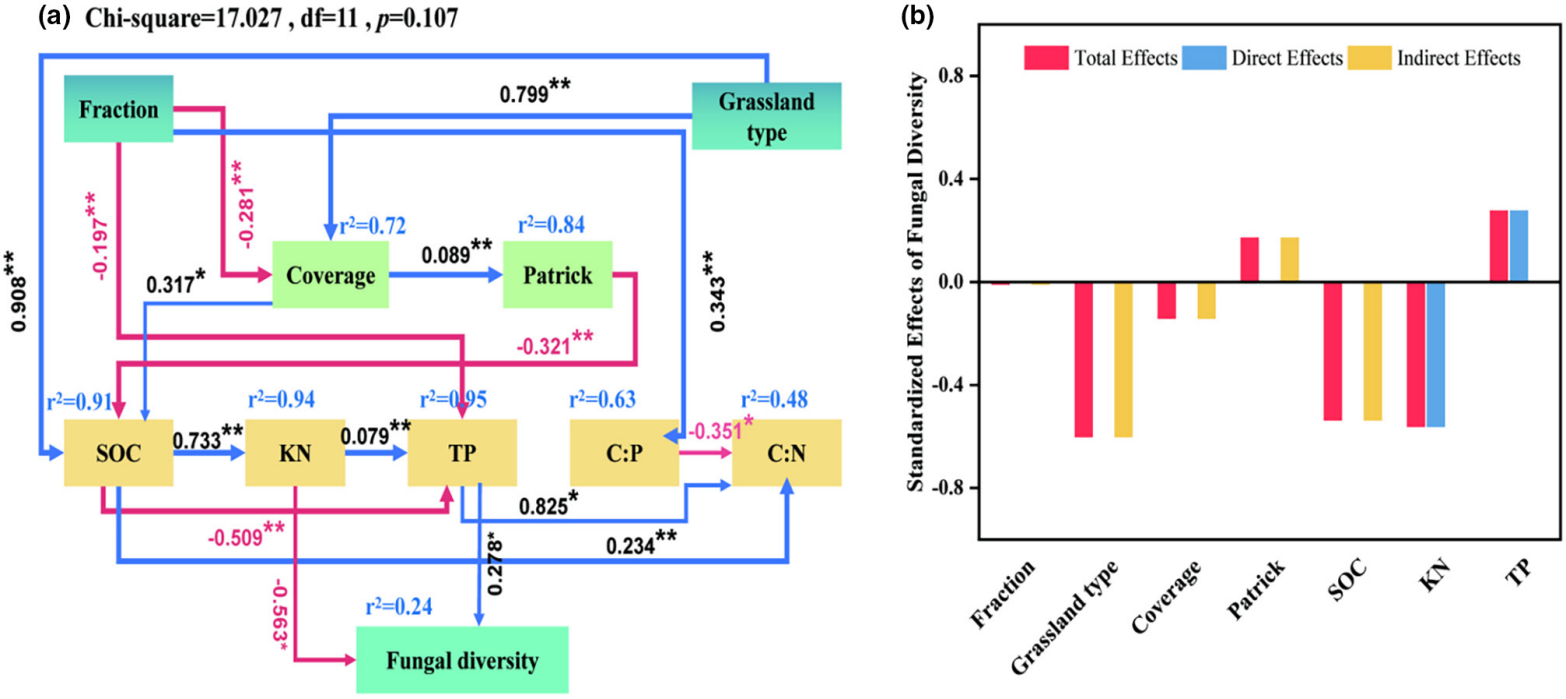
(a) Structural equation modeling exploring the direct and indirect effects of the fraction (grazing and grazing exclusion), grassland type, plant index (coverage and Patrick index), and soil chemical properties on fungal diversity and (b) standardized effects of fraction, grassland type, plant index, and soil chemical properties on fungal diversity. Line thickness indicates the correlation strength: the thicker the line, the stronger the correlation. The red and blue arrows indicate negative and positive relationships, respectively. The numbers adjacent to the arrows represent the standardized path coefficients. The proportion of variance explained (*R* ^2^) appears alongside each response variable in the model.

Structure equation modeling (SEM) analysis showed that soil kjeldahl nitrogen and total soil phosphorus had direct effects on fungal community diversity, while grazing exclusion, grassland type, and SOC had indirect effects on fungal community diversity. Fraction had a significant effect on fungal community diversity by directly affecting total soil phosphorus (*p* < .05) and grassland type had a significant effect on fungal community diversity by directly affecting SOC, which in turn affected KN and TP (*p* < .05). These variables explained approximately 24% of the variation in fungal community diversity (Figure 7a). In addition, a combination of direct and indirect effects revealed that grassland type was the most important factor influencing changes in fungal community diversity, and in terms of plant characteristics and soil nutrients, vegetation cover and soil kjeldahl nitrogen were the main factors influencing fungal diversity (Figure 7b).

## 4 DISCUSSION

### 4.1 Effect of grazing exclusion on soil physicochemical properties in different grassland types

Irrespective of whether grazing exclusion can change the diversity and composition of soil fungal communities, soil chemistry is the dominant factor (Li et al., 2019; Qin et al., 2021). There is growing evidence that fungal communities are more sensitive than bacterial communities to carbon and nitrogen sources (Zhang et al., 2018). Our study found that C:N did not differ significantly among the three grassland types in the 0–5 cm soils before grazing exclusion, probably because grazing can promote root secretions and fallen leaf decomposition through trampling, which can increase the amount of C and N entering the soil and keep the soil C and N in dynamic equilibrium (Bi et al., 2018). In the 5–10 cm soil layer, whereas the C:N was significantly higher in temperate deserts than in temperate steppes and mountain meadows after grazing exclusion, the interaction of exclusion and grassland type significantly altered the C:N, indicating that grazing exclusion reduced the soil C and N pools (Sun et al., 2020), and this result was prominent in steppes and meadows. In addition, we found that grazing exclusion significantly increased SOC in the 0–5 cm soil layer of temperate steppes and the 5–10 cm soil layer of mountain meadows; moreover, grazing exclusion significantly increased SWC in the 0–5 cm soil layer of temperate steppes and the 5–10 cm soil layer of mountain meadows due to higher soil fertility associated with good plant cover and greater apoplastic accumulation (Jing et al., 2014; Marzaioli et al., 2010). Aboveground biomass has a direct effect on apoplastic accumulation, which increases soil moisture. This explains the increase in SWC in both temperate steppes and mountain meadows. Furthermore, apoplastic decomposes more rapidly in moist soils, and large amounts of decomposing apoplastic can increase soil nutrients (Jing et al., 2014).

Furthermore, soil TP showed a close correlation with temperate deserts, and grazing exclusion significantly increased the TP content in temperate deserts, which is inconsistent with previous studies (Yang, Sun, et al., 2018; Yang, Zhang, et al., 2018). This difference may be because soil nutrients still had not returned to their original levels after 9 years of grazing exclusion but were in an unstable and fluctuating state. This may be related to the change in the main vegetation types on the surface, the rate of decomposition, and the amount of nutrient uptake and return by plant roots. The effects of grazing exclusion on pH, C:N, C:P, and N:P in the three grasslands were not significant regardless of the soil stratum. The reasons for this phenomenon may be the removal of grazing pressure after grazing exclusion in degraded grasslands, the disappearance of conditions that maintained the spatial pattern of soil nutrients or vegetation, an increase in the spatial heterogeneity of soil nutrients, and an increase in the proportion of stochastic factors, making the research results more stochastic and the study results appear to be different (David, 1988). Additionally, regional differences in the soil itself may also be responsible for this difference (Yang et al., 2022).

### 4.2 Effects of grazing exclusion on the composition and diversity of soil fungi

Our results go further than the commonly reported differences in fungal composition between grassland types (Wang et al., 2021; Yang et al., 2022; Zhang et al., 2010). In different grassland types, the response of fungal composition to grazing exclusion was basically the same. It is worth mentioning that the changes in Mortierellomycota, which we found to be significantly lower after grazing exclusion in the 0–5 soil layer in mountain meadows and highly significant in the 5–10 soil layer in temperate deserts. This is consistent with previous findings that grazing exclusion is more favorable to the relative abundance of Mortierellomycota (Wang et al., 2023), as a relatively unique group in the soil, it may play a role in promoting material circulation in grazing exclusion grasslands with low nutrient content (Dai et al., 2021). This answered the second scientific question of the study. Consistent with previous results (Maestre et al., 2015; Ren et al., 2017), we found that at the phylum level, *Ascomycota* and *Basidiomycota* were the dominant pregrazing exclusion fungi in all three grassland types. After the grazing exclusion, *Ascomycota* decreased by 14.72% and 9.74% in temperate deserts and mountain meadows, respectively, while it increased by 4.82% in temperate steppes. In contrast to *Ascomycota*, the *Basidiomycota* increased by 238.8% and 47.68% in temperate deserts and mountain meadows, respectively, while it decreased by 23.12% in temperate steppes. This result may be due to the higher soil pH in temperate deserts, the acidic soils of temperate steppes and mountain meadows, and the positive responses of *Ascomycota* and *Basidiomycota* fungi to changes in soil pH, with a higher abundance of *Ascomycota* in soils with higher pH (Tedersoo et al., 2014). The correlation analysis results also confirmed the above view, and the results showed that pH was significantly positively correlated with *Dothideomycetes* (a class of *Ascomycota* subphylum), which also explained the higher relative abundance of *Ascomycota* in temperate deserts. Additionally, studies of soil fungi have found that the soil fungal community has a strong spatial structure along the ecological gradients, and some fungi can dominate many environments. Wind‐dispersed Ascomycota taxa dominated the soil fungal communities, followed by Basidiomycota and other fungi (Bucbe et al., 2009; Egidi et al., 2019). Next, significant differences were found among the three grassland types for the *Glomeromycota* and *Mucoromycota*, and it was found that the *Mortierellomycota* was not influenced by either grazing exclusion or grassland type, but the interaction between the two had a significant effect on the *Mortierellomycota*. It has been shown that the activity of *Glomeromycota* is reduced in highly acidic environments and is not efficient at taking up nitrogen or phosphorus from organic matter and converting it to inorganic components for plant use; eventually, plants gradually form new symbiotic relationships with other organic matter mycorrhizal fungi and replace the *Glomeromycota* (Smith & Read, 2008). In this study, the organic matter content of the temperate desert soil was low and the relative abundance of *Glomeromycota* was lower than that in the other two types of grassland. This study validates the findings of most studies regarding the relationship between *Glomeromycota* and soil physicochemical properties and supports previous studies suggesting that soil organic matter content has a suppressive effect on *Glomeromycota* (Pellissier et al., 2015; Smith & Read, 2008). Furthermore, at the class level, the dominant fungi in the three grassland types before and after exclusion included Dothideomycetes, Archaeorhizomycetes, Sordariomycetes, and Agaricomycetes, while in the temperate desert, Archaeorhizomycetes almost disappeared. This result indicates that fungi display incredible functional diversity at the class levels. Some fungi, which were just as sensitive as bacteria, disappeared (and were replaced by something else) in response to the treatment or grassland type. When some taxa disappear, other taxa are well adapted to the new niches that are created. The different responses of various fungal classes to environmental change reflect the role of environmental niche separation in selecting fungal communities (Pellissier et al., 2015).

Unlike the effect of grazing exclusion on soil fungal composition, grazing exclusion, grassland type, and the interaction between the two did not significantly alter soil fungal α‐diversity in either the 0–5 cm or the 5–10 cm soils, similar to previous findings (Ding et al., 2020; Hao et al., 2020; Yang et al., 2019). The response of fungal α‐diversity to grazing exclusion and grassland type was not significant, essentially meaning that there were no significant differences in the taxonomic richness among the sites (Cheng et al., 2016; Zhang et al., 2018). These results were consistent with previous findings that soil fungi were more stable than bacteria (Hamonts et al., 2017; Wang et al., 2019). Although there was no effect on fungal α‐diversity, we found that the interaction of grazing exclusion and grassland type significantly altered fungal β‐diversity. Changes in the β‐diversity of fungi may indicate the ability of many soil microorganisms to colonize the inter‐rhizosphere, where root biomass and plant C:N ratios may influence the utilization of inter‐rhizosphere resources by altering root turnover or root secretions, which may in turn strongly alter the composition of the microbial community (Chase, 2010; Philippot et al., 2013). Moreover, other studies have detected significant effects of grazing exclusion on soil fungal β‐diversity (Chen et al., 2020). In the current study, the strong link between soil microbial diversity and grazing exclusion and grassland type can be explained by the fact that microbial communities are often directly influenced by aboveground plant biomass and community structure (de Vries et al., 2012). For example, grazing exclusion increases aboveground plant biomass and apoplastic content, thus increasing fungal diversity (Hamonts et al., 2017). The increase in species richness induced by grazing exclusion suggests that high plant diversity results in a greater variety of organic matter ultimately flowing into the subsurface, thereby generating more ecological niches for use by different species of microorganisms and altering the diversity of microbial communities (Brodie et al., 2003; Lodge, 1997). The results showed that under the influence of different grazing methods and different grassland types, the soil fungal community composition changed, and the difference in β‐diversity was significant, but the change in soil fungal α‐diversity was not significant, which answered the first scientific question of this study.

### 4.3 Potential drivers of soil fungal community diversity

SEM analysis showed that grazing exclusion had a significant effect on fungal community diversity by directly affecting total soil phosphorus, and that grassland type had a significant effect on fungal community diversity by directly affecting SOC, which in turn affected KN and TP. In line with our findings, other studies have also shown that soil TP and KN are closely related to soil fungal diversity (Lauber et al., 2008; Lindahl & Tunlid, 2015). In contrast, a recent experiment showed that soil fungal abundance and diversity differed between grassland habitats, but that this difference was not correlated with geographical distance, and that certain environmental factors, including climate, soil pH, nitrogen, and phosphorus, were the main influences affecting the distribution, abundance, and diversity of soil fungal communities (Pellissier et al., 2015). In our study, a combination of direct and indirect effects revealed that grassland type was the most important factor influencing changes in fungal community diversity, confirming that natural environments in different regions affect soil fungal diversity, and that harsh environments, such as drought, reduce fungal diversity and abundance (Maestre et al., 2015).In addition, we found that soil KN was the most important predictor of changes in fungal community diversity, and the strong association between soil KN and fungal diversity was justified because KN provides the main energy source for soil microorganisms (Lindahl & Tunlid, 2015) SOC has an indirect effect on fungal community diversity, which is similar to previous findings. SOC provides nutrients and energy for plant growth and soil microbial life (Li et al., 2006), and its content reflects the effectiveness of limiting resources in the soil, and it is the nutrient “source” for the growth of soil fungal communities, and the level of its content affects the structure and diversity of soil fungal communities. In conclusion, the results of the study answered the third scientific question: grassland type is the most important factor influencing changes in the diversity of fungal communities, while in terms of plant characteristics and soil nutrients, vegetation cover and soil kjeldahl nitrogen are the main factors influencing fungal diversity. However, this paper still has some limitations, and our results did not take into account seasonal and annual differences in soil fungal communities, which need to be analyzed further in depth. Future experimental studies can explore the potential effects of climate change on soil fungal community diversity and microbial assembly processes in different grazing ban years or seasons, and provide more theoretical basis for clearing the blind spots in this field.

## 5 CONCLUSIONS

Grazing exclusion is one of the most commonly used measures to rehabilitate degraded grasslands globally. Therefore, assessing its ecological impact is important for grassland conservation and biodiversity management. As revealed by the data from our study, the composition of soil fungal communities differed between grassland types More importantly, the dominant fungal taxa such as *Glomeromycetes*, *Orbiliomycetes*, *Tremellomycetes*, *Ustilaginomycetes*, *Eurotiomycetes*, *Orbiliomycetes*, *Dothideomycetes*, and *Archaeorhizomycetes* shed new light on the involvement of soil microbes in different grassland types. The response of fungal composition to grazing exclusion was basically the same in the different grassland types, except for the *Mortierellomycota*. Under the influence of both grazing exclusion and grassland type, there was no significant change in soil fungal α‐diversity, but there were significant differences in fungal β‐diversity. Grassland type was the most important factor influencing changes in fungal community diversity, and vegetation cover and soil kjeldahl nitrogen were the main factors influencing fungal diversity. Our research provides a long‐term perspective for better understanding and managing different grasslands, as well as a better scientific basis for future research on grass–soil–microbe interactions.

## AUTHOR CONTRIBUTIONS

**Shijie Zhou:** Formal analysis (equal); investigation (equal); methodology (equal); visualization (lead); writing – original draft (lead); writing – review and editing (lead). **Yiqiang Dong:** Funding acquisition (lead); investigation (equal); project administration (lead); resources (equal); supervision (lead). **Helong Yang:** Data curation (equal); resources (equal); writing – review and editing (equal). **Suwen Yang:** Data curation (equal); software (equal); writing – review and editing (equal). **Asitaiken Julihaiti:** Data curation (equal); resources (equal); software (equal). **Zeyu Liu:** Data curation (equal); software (equal). **Tingting Nie:** Data curation (equal); resources (equal). **Anjing Jiang:** Data curation (equal); resources (equal); software (equal). **Yue Wu:** Data curation (equal); resources (equal); software (equal). **Shazhou An:** Funding acquisition (equal); investigation (equal); resources (equal); supervision (equal); writing – original draft (equal); writing – review and editing (equal).

## CONFLICT OF INTEREST STATEMENT

The authors declare that they have no competing interests.

## Supporting information


Appendix S1.

## Data Availability

Hereby affirm that primary data including total data will be deposited in the Dryad Repository when the paper is accepted. Dryad. https://doi.org/10.5061/dryad.dbrv15f5p. The data that support the findings of this study are available from the corresponding author upon reasonable request. The data will be stored in https://datadryad.org/stash/share/Uu9x2‐0o0c0CEOaAwGRA3yd8bbVIDCZVHN1qADDpZAc.
