# Supplementary material for: Effects of grazing exclusion on soil properties, fungal community structure, and diversity in different grassland types
Source: Ecol Evol. 2024 Mar 1;14(3):e11056. doi: 10.1002/ece3.11056 (PMC10905231; doi:10.1002/ece3.11056)
Supplement: Supplementary file 1 — Appendix S1. [file ECE3-14-e11056-s001.docx]

**Appendice**

**Supplementary Table S1** Vegetation characteristics as affected by grazing exclusion and grassland type.

| Index | Factor | Sum sq | Df | F | P | Significance |
| --- | --- | --- | --- | --- | --- | --- |
| Height | Fraction | 413.808 | 1 | 3.24300 | 0.078 | / |
|  | Grassland type | 39916.54 | 2 | 156.427 | <0.001 | ** |
|  | Fraction*Grassland type | 998.447 | 2 | 3.913 | 0.027 | * |
| Coverage | Fraction | 13781.473 | 1 | 72.02300 | <0.001 | ** |
|  | Grassland type | 139491.244 | 2 | 364.495 | <0.001 | ** |
|  | Fraction*Grassland type | 11806.437 | 2 | 30.851 | <0.001 | ** |
| Density | Fraction | 22716.876 | 1 | 0.784 | 0.38 | / |
|  | Grassland type | 2367298.69 | 2 | 40.853 | <0.001 | ** |
|  | Fraction*Grassland type | 141661.519 | 2 | 2.445 | 0.097 | / |
| Biomass | Fraction | 4038.069 | 1 | 8.95 | 0.004 | * |
|  | Grassland type | 41503.858 | 2 | 45.995 | <0.001 | ** |
|  | Fraction*Grassland type | 5340.404 | 2 | 5.918 | 0.005 | * |
| Patrick | Fraction | 56.019 | 1 | 33.518 | <0.001 | ** |
|  | Grassland type | 972.111 | 2 | 290.825 | <0.001 | ** |
|  | Fraction*Grassland type | 62.481 | 2 | 18.693 | <0.001 | ** |
| Shannon | Fraction | 0.745 | 1 | 13.759 | 0.001 | * |
|  | Grassland type | 23.559 | 2 | 217.526 | <0.001 | ** |
|  | Fraction*Grassland type | 3.526 | 2 | 32.554 | <0.001 | ** |
| Simpson | Fraction | 0.012 | 1 | 0.827 | 0.368 | / |
|  | Grassland type | 1.675 | 2 | 57.137 | <0.001 | ** |
|  | Fraction*Grassland type | 0.53 | 2 | 18.062 | <0.001 | ** |
| Pielou | Fraction | 0.015 | 1 | 0.353 | 0.555 | / |
|  | Grassland type | 0.211 | 2 | 2.488 | 0.094 | / |
|  | Fraction*Grassland type | 0.572 | 2 | 6.733 | 0.003 | * |

Fraction represents grazing exclusion and grazing; Grassland type represents temperate desert、temperate steppe and mountain meadow.

The interaction of grazing exclusion and grassland type significantly altered TN and N:P *(P* < 0.05; Supplementary Table 2). Soil physicochemical properties were all highly significantly influenced by different grassland types in general compared to grazing exclusion, while grazing exclusion, grassland type and the interaction between the two did not significantly alter C:N.

**Supplementary Table S2** Soil physicochemical properties as affected by grazing exclusion and grassland type in the 0-5 cm soil layer.

| Index | Factor | Sum sq | Df | F | P | Significance |
| --- | --- | --- | --- | --- | --- | --- |
| pH | Fraction | 0.00005 | 1 | 0.00100 | 0.97400 | / |
|  | Grassland type | 29.703 | 2 | 331.255 | <0.001 | ** |
|  | Fraction*Grassland type | 0.118 | 2 | 1.316 | 0.304 | / |
| SWC | Fraction | 0.002 | 1 | 3.67400 | 0.07900 | / |
|  | Grassland type | 0.113 | 2 | 95.773 | <0.001 | ** |
|  | Fraction*Grassland type | 0.003 | 2 | 2.49 | 0.125 | / |
| BD | Fraction | 0.051 | 1 | 9.244 | 0.01 | * |
|  | Grassland type | 1.85 | 2 | 168.454 | <0.001 | ** |
|  | Fraction*Grassland type | 0.002 | 2 | 0.173 | 0.843 | / |
| SOC | Fraction | 196.482 | 1 | 1.244 | 0.287 | / |
|  | Grassland type | 37464.299 | 2 | 118.62 | <0.001 | ** |
|  | Fraction*Grassland type | 514.061 | 2 | 1.628 | 0.237 | / |
| KN | Fraction | 0.773 | 1 | 0.964 | 0.346 | / |
|  | Grassland type | 336.497 | 2 | 209.709 | <0.001 | ** |
|  | Fraction*Grassland type | 13.536 | 2 | 8.436 | 0.005 | * |
| TP | Fraction | 0.027 | 1 | 4.047 | 0.067 | / |
|  | Grassland type | 0.703 | 2 | 52.421 | <0.001 | ** |
|  | Fraction*Grassland type | 0.044 | 2 | 3.259 | 0.074 | / |
| C:N | Fraction | 13.904 | 1 | 0.579 | 0.461 | / |
|  | Grassland type | 29.729 | 2 | 0.619 | 0.555 | / |
|  | Fraction*Grassland type | 30.205 | 2 | 0.629 | 0.55 | / |
| C:P | Fraction | 39.635 | 1 | 0.156 | 0.7 | / |
|  | Grassland type | 43205.924 | 2 | 84.826 | <0.001 | ** |
|  | Fraction*Grassland type | 4.201 | 2 | 0.008 | 0.992 | / |
| N:P | Fraction | 0.271 | 1 | 1.123 | 0.31 | / |
|  | Grassland type | 368.917 | 2 | 763.417 | <0.001 | ** |
|  | Fraction*Grassland type | 4.814 | 2 | 9.963 | 0.003 | * |

Fraction represents grazing exclusion and grazing; Grassland type represents temperate desert、temperate steppe and mountain meadow. SWC, soil water content; BD, bulk density; SOC, soil organic carbon; KN, Kjeldahl nitrogen; TP, total phosphorus; C:N, carbon:nitrogen; C:P, carbon:phosphorus; N:P, nitrogen:phosphorus.

The interaction of grazing exclusion and grassland type significantly altered BD, SOC, TP and C:N (*P* < 0.05; Supplementary Table 3).

**Supplementary Table S3** Soil physicochemical properties as affected by grazing exclusion and grassland type in the 5-10 cm soil layer.

| Index | Factor | Sum sq | Df | F | P | Significance |
| --- | --- | --- | --- | --- | --- | --- |
| pH | Fraction | 0.019 | 1 | 0.31700 | 0.58400 | / |
|  | Grassland type | 36.043 | 2 | 305.539 | <0.001 | ** |
|  | Fraction*Grassland type | 0.032 | 2 | 0.275 | 0.764 | / |
| SWC | Fraction | 0.001 | 1 | 2.47500 | 0.14200 | / |
|  | Grassland type | 0.109 | 2 | 192.509 | <0.001 | ** |
|  | Fraction*Grassland type | 0.001 | 2 | 2.631 | 0.113 | / |
| BK | Fraction | 0.052 | 1 | 19.265 | 0.001 | * |
|  | Grassland type | 1.513 | 2 | 278.447 | <0.001 | ** |
|  | Fraction*Grassland type | 0.024 | 2 | 4.497 | 0.035 | * |
| SOC | Fraction | 10.58 | 1 | 0.235 | 0.637 | / |
|  | Grassland type | 21243.061 | 2 | 235.674 | <0.001 | ** |
|  | Fraction*Grassland type | 681.732 | 2 | 7.563 | 0.007 | * |
| KN | Fraction | 0.702 | 1 | 0.571 | 0.464 | / |
|  | Grassland type | 225.469 | 2 | 91.649 | <0.001 | ** |
|  | Fraction*Grassland type | 9.594 | 2 | 3.9 | 0.05 | / |
| TP | Fraction | 0.046 | 1 | 6.335 | 0.027 | * |
|  | Grassland type | 0.496 | 2 | 34.063 | <0.001 | ** |
|  | Fraction*Grassland type | 0.084 | 2 | 5.786 | 0.017 | * |
| C:N | Fraction | 40.081 | 1 | 2.039 | 0.179 | / |
|  | Grassland type | 260.24 | 2 | 6.62 | 0.012 | * |
|  | Fraction*Grassland type | 231.203 | 2 | 5.881 | 0.017 | * |
| C:P | Fraction | 323.851 | 1 | 1.814 | 0.203 | / |
|  | Grassland type | 29164.376 | 2 | 81.695 | <0.001 | ** |
|  | Fraction*Grassland type | 180.284 | 2 | 0.505 | 0.616 | / |
| N:P | Fraction | 0.02 | 1 | 0.042 | 0.841 | / |
|  | Grassland type | 293.325 | 2 | 306.298 | <0.001 | ** |
|  | Fraction*Grassland type | 0.873 | 2 | 0.912 | 0.428 | / |

Fraction represents grazing exclusion and grazing; Grassland type represents temperate desert、temperate steppe and mountain meadow. SWC, soil water content; BD, bulk density; SOC, soil organic carbon; KN, Kjeldahl nitrogen; TP, total phosphorus; C:N, carbon:nitrogen; C:P, carbon:phosphorus; N:P, nitrogen:phosphorus.

According to supplementary Figure1, in the temperate desert, grazing exclusion increased the number of OTUs in the 0-5 cm and 5-10 cm soil layers by 96.64% and 32.59%, respectively, and the total number of OTUs in the four treatments was 172. In temperate steppe, grazing exclusion decreased the OTUs quantity in 0-5 cm soil layer by 15.01% and 13.96% in 5-10 cm soil layer, respectively, and the total OTUs number in the four treatments was 116. In the mountain meadow, grazing exclusion decreased the OTUs quantity of 0-5 cm soil layer by 11.06% and increased the OTUs quantity of 5-10 cm soil layer by 15.34%, respectively, and the total OTUs number of the four treatments was 131.

**Supplementary Fig1.** Venn diagram of soil fungi community under different soil layers and grazing exclusion. Figure A-C represents Temperate desert, Temperate grassland and Mountain meadow respectively, and different colors represent different treatments and soil layers respectively.


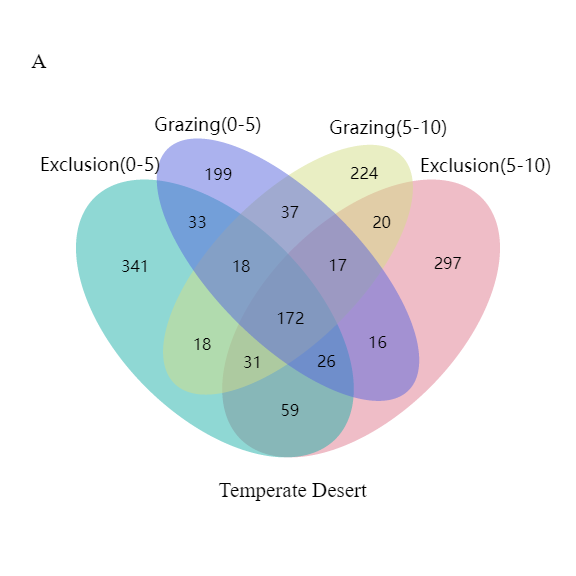

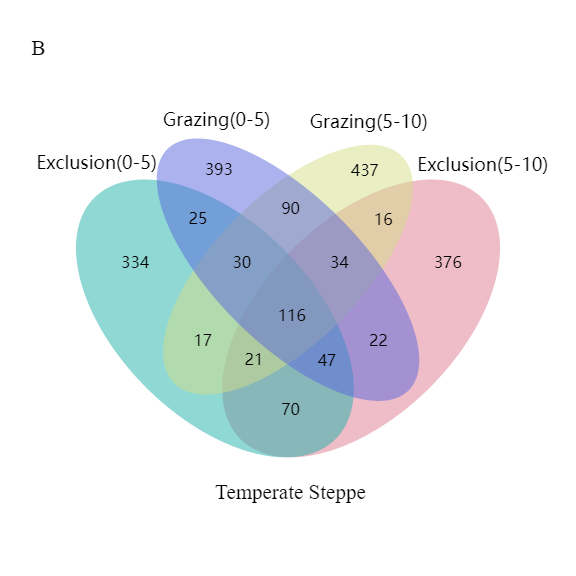

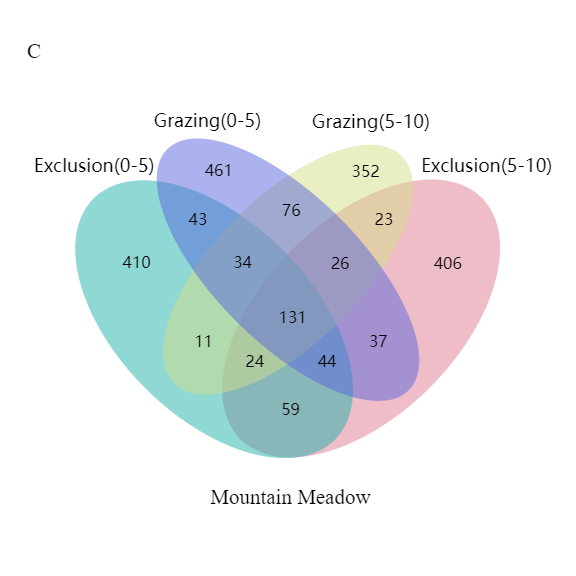


Significant differences were found between the three grassland types for the *Glomeromycota* and *Mucoromycota* (P<0.05; Supplementary Table 4). The interaction between grazing exclusion and grassland type had no significant effect on the other fungi.

**Supplementary Table S4** Fungal community phylum level as affected by grazing exclusion and grassland type in the 0-5 cm soil layer.

| Fungi | Factor | F | P |
| --- | --- | --- | --- |
| Ascomycota | Fraction | 0.758 | 0.401 |
|  | Grassland type | 1.183 | 0.340 |
|  | Fraction*Grassland type | 0.596 | 0.567 |
| Basidiomycota | Fraction | 0.742 | 0.406 |
|  | Grassland type | 0.764 | 0.487 |
|  | Fraction*Grassland type | 0.580 | 0.575 |
| Glomeromycota | Fraction | 0.261 | 0.619 |
|  | Grassland type | 5.504 | 0.02* |
|  | Fraction*Grassland type | 0.669 | 0.530 |
| Chytridiomycota | Fraction | 0.441 | 0.519 |
|  | Grassland type | 0.770 | 0.485 |
|  | Fraction*Grassland type | 0.024 | 0.977 |
| Olpidiomycota | Fraction | 0.079 | 0.784 |
|  | Grassland type | 2.449 | 0.128 |
|  | Fraction*Grassland type | 0.724 | 0.505 |
| Rozellomycota | Fraction | 1.861 | 0.198 |
|  | Grassland type | 2.023 | 0.175 |
|  | Fraction*Grassland type | 0.997 | 0.398 |
| Mucoromycota | Fraction | 3.223 | 0.098 |
|  | Grassland type | 9.587 | 0.003* |
|  | Fraction*Grassland type | 3.223 | 0.076 |
| Mortierellomycota | Fraction | 0.023 | 0.882 |
|  | Grassland type | 2.971 | 0.090 |
|  | Fraction*Grassland type | 1.586 | 0.245 |
| Blastocladiomycota | Fraction | 2.807 | 0.120 |
|  | Grassland type | 3.726 | 0.055 |
|  | Fraction*Grassland type | 0.784 | 0.478 |
| Entorrhizomycota | Fraction | 0.767 | 0.398 |
|  | Grassland type | 1.233 | 0.326 |
|  | Fraction*Grassland type | 0.767 | 0.486 |
| Kickxellomycota | Fraction | 0.008 | 0.929 |
|  | Grassland type | 2.186 | 0.155 |
|  | Fraction*Grassland type | 0.211 | 0.812 |
| Zoopagomycota | Fraction | 1.000 | 0.337 |
|  | Grassland type | 1.000 | 0.397 |
|  | Fraction*Grassland type | 1.000 | 0.397 |
| Monoblepharomycota | Fraction | 0.000 | 1.000 |
|  | Grassland type | 1.996 | 0.178 |
|  | Fraction*Grassland type | 0.000 | 1.000 |

Fraction represents grazing exclusion and grazing; Grassland type represents temperate desert、temperate steppe and mountain meadow.

The analysis revealed that the *Mortierellomycota* was not affected by either grazing exclusion or grassland type, but the interaction between the two had a significant effect on the *Mortierellomycota* (*P* < 0.05; Supplementary Table 5). In addition, grassland type significantly influenced the *Blastocladiomycota* (*P* < 0.05), whereas grazing exclusion had no significant effect on any of the fungi measured (P > 0.05).

**Supplementary Table S5** Fungal community phylum level as affected by grazing exclusion and grassland type in the 5-10 cm soil layer.

| Fungi | Factor | F | P |
| --- | --- | --- | --- |
| Ascomycota | Fraction | 0.023 | 0.882 |
|  | Grassland type | 1.374 | 0.290 |
|  | Fraction*Grassland type | 0.999 | 0.397 |
| Basidiomycota | Fraction | 0.000 | 0.986 |
|  | Grassland type | 0.899 | 0.433 |
|  | Fraction*Grassland type | 1.217 | 0.330 |
| Glomeromycota | Fraction | 1.499 | 0.244 |
|  | Grassland type | 2.853 | 0.097 |
|  | Fraction*Grassland type | 0.654 | 0.538 |
| Chytridiomycota | Fraction | 1.268 | 0.282 |
|  | Grassland type | 1.433 | 0.277 |
|  | Fraction*Grassland type | 1.302 | 0.308 |
| Olpidiomycota | Fraction | 2.298 | 0.155 |
|  | Grassland type | 0.817 | 0.465 |
|  | Fraction*Grassland type | 0.569 | 0.580 |
| Rozellomycota | Fraction | 0.033 | 0.860 |
|  | Grassland type | 0.195 | 0.825 |
|  | Fraction*Grassland type | 1.727 | 0.219 |
| Mucoromycota | Fraction | 0.208 | 0.657 |
|  | Grassland type | 0.710 | 0.511 |
|  | Fraction*Grassland type | 1.676 | 0.228 |
| Mortierellomycota | Fraction | 1.969 | 0.186 |
|  | Grassland type | 3.670 | 0.057 |
|  | Fraction*Grassland type | 6.345 | 0.013* |
| Blastocladiomycota | Fraction | 0.496 | 0.495 |
|  | Grassland type | 4.292 | 0.039* |
|  | Fraction*Grassland type | 0.496 | 0.621 |
| Entorrhizomycota | Fraction | 1.000 | 0.337 |
|  | Grassland type | 1.000 | 0.397 |
|  | Fraction*Grassland type | 1.000 | 0.397 |
| Kickxellomycota | Fraction | 0.000 | 1.000 |
|  | Grassland type | 1.646 | 0.234 |
|  | Fraction*Grassland type | 1.215 | 0.331 |
| Basidiobolomycota | Fraction | 1.000 | 0.337 |
|  | Grassland type | 1.000 | 0.397 |
|  | Fraction*Grassland type | 1.000 | 0.397 |

Fraction represents grazing exclusion and grazing; Grassland type represents temperate desert、temperate steppe and mountain meadow.

Grassland type had a significant effect on *Glomeromycetes*, *Orbiliomycetes* and *Tremellomycetes* (P < 0.05; Supplementary Table 6).

**Supplementary Table S6** Fungal community phyla level as affected by grazing exclusion and grassland type in the 0-5 cm soil layer.

| Fungi | Factor | F | P |
| --- | --- | --- | --- |
| Other | Fraction | 0.185 | 0.694 |
|  | Grassland type | 2.429 | 0.192 |
|  | Fraction*Grassland type | 1.302 | 0.337 |
| Dothideomycetes | Fraction | 1.802 | 0.204 |
|  | Grassland type | 59.103 | 0.000** |
|  | Fraction*Grassland type | 0.544 | 0.594 |
| Archaeorhizomycetes | Fraction | 0.849 | 0.375 |
|  | Grassland type | 21.933 | 0.000** |
|  | Fraction*Grassland type | 0.418 | 0.667 |
| Sordariomycetes | Fraction | 1.494 | 0.245 |
|  | Grassland type | 2.422 | 0.131 |
|  | Fraction*Grassland type | 0.188 | 0.831 |
| Agaricomycetes | Fraction | 0.066 | 0.801 |
|  | Grassland type | 3.545 | 0.062 |
|  | Fraction*Grassland type | 0.523 | 0.606 |
| Eurotiomycetes | Fraction | 0.413 | 0.533 |
|  | Grassland type | 3.004 | 0.088 |
|  | Fraction*Grassland type | 0.662 | 0.534 |
| Glomeromycetes | Fraction | 0.134 | 0.721 |
|  | Grassland type | 7.719 | 0.007* |
|  | Fraction*Grassland type | 0.906 | 0.430 |
| Leotiomycetes | Fraction | 0.269 | 0.614 |
|  | Grassland type | 1.516 | 0.259 |
|  | Fraction*Grassland type | 0.083 | 0.921 |
| Pezizomycetes | Fraction | 0.786 | 0.393 |
|  | Grassland type | 0.791 | 0.476 |
|  | Fraction*Grassland type | 1.857 | 0.198 |
| Orbiliomycetes | Fraction | 1.105 | 0.314 |
|  | Grassland type | 7.587 | 0.007* |
|  | Fraction*Grassland type | 0.406 | 0.675 |
| Tremellomycetes | Fraction | 0.067 | 0.800 |
|  | Grassland type | 5.663 | 0.019* |
|  | Fraction*Grassland type | 0.186 | 0.832 |
| Saccharomycetes | Fraction | 0.946 | 0.350 |
|  | Grassland type | 0.926 | 0.423 |
|  | Fraction*Grassland type | 1.049 | 0.380 |
| Rhizophlyctidomycetes | Fraction | 1.096 | 0.316 |
|  | Grassland type | 1.035 | 0.385 |
|  | Fraction*Grassland type | 0.956 | 0.412 |
| Ustilaginomycetes | Fraction | 0.710 | 0.416 |
|  | Grassland type | 1.507 | 0.261 |
|  | Fraction*Grassland type | 0.710 | 0.511 |
| Spizellomycetes | Fraction | 0.036 | 0.852 |
|  | Grassland type | 0.792 | 0.475 |
|  | Fraction*Grassland type | 0.815 | 0.466 |
| Exobasidiomycetes | Fraction | 0.750 | 0.404 |
|  | Grassland type | 0.866 | 0.445 |
|  | Fraction*Grassland type | 1.125 | 0.357 |
| Olpidiomycetes | Fraction | 0.074 | 0.790 |
|  | Grassland type | 2.756 | 0.104 |
|  | Fraction*Grassland type | 0.703 | 0.514 |
| Geoglossomycetes | Fraction | 1.085 | 0.318 |
|  | Grassland type | 1.139 | 0.352 |
|  | Fraction*Grassland type | 1.017 | 0.391 |
| Lecanoromycetes | Fraction | 3.159 | 0.101 |
|  | Grassland type | 2.368 | 0.136 |
|  | Fraction*Grassland type | 3.232 | 0.075 |
| Geminibasidiomycetes | Fraction | 1.000 | 0.337 |
|  | Grassland type | 1.000 | 0.397 |
|  | Fraction*Grassland type | 1.000 | 0.397 |

Fraction represents grazing exclusion and grazing; Grassland type represents temperate desert、temperate steppe and mountain meadow.

The interaction of grazing exclusion, grassland type and both significantly altered Sordariomycetes and Pezizomycetes (P < 0.05; Supplementary Table 7).

**Supplementary Table S7** Fungal community phyla level as affected by grazing exclusion and grassland type in the 5-10 cm soil layer.

| Fungi | Factor | F | P |
| --- | --- | --- | --- |
| Other | Fraction | 1.060 | 0.338 |
|  | Grassland type | 3.671 | 0.127 |
|  | Fraction*Grassland type | 0.773 | 0.487 |
| Dothideomycetes | Fraction | 2.093 | 0.174 |
|  | Grassland type | 15.439 | 0.000** |
|  | Fraction*Grassland type | 2.548 | 0.120 |
| Archaeorhizomycetes | Fraction | 2.105 | 0.172 |
|  | Grassland type | 45.338 | 0.000** |
|  | Fraction*Grassland type | 0.559 | 0.586 |
| Sordariomycetes | Fraction | 5.136 | 0.043* |
|  | Grassland type | 10.071 | 0.003* |
|  | Fraction*Grassland type | 4.106 | 0.044* |
| Agaricomycetes | Fraction | 0.100 | 0.758 |
|  | Grassland type | 2.402 | 0.133 |
|  | Fraction*Grassland type | 1.084 | 0.369 |
| Eurotiomycetes | Fraction | 0.521 | 0.484 |
|  | Grassland type | 5.260 | 0.023* |
|  | Fraction*Grassland type | 0.637 | 0.546 |
| Glomeromycetes | Fraction | 1.712 | 0.215 |
|  | Grassland type | 2.531 | 0.121 |
|  | Fraction*Grassland type | 0.428 | 0.662 |
| Leotiomycetes | Fraction | 0.208 | 0.656 |
|  | Grassland type | 1.991 | 0.179 |
|  | Fraction*Grassland type | 0.255 | 0.779 |
| Pezizomycetes | Fraction | 8.044 | 0.015* |
|  | Grassland type | 9.622 | 0.003* |
|  | Fraction*Grassland type | 10.436 | 0.002* |
| Orbiliomycetes | Fraction | 0.084 | 0.777 |
|  | Grassland type | 4.660 | 0.032* |
|  | Fraction*Grassland type | 1.121 | 0.358 |
| Tremellomycetes | Fraction | 0.637 | 0.440 |
|  | Grassland type | 3.783 | 0.053 |
|  | Fraction*Grassland type | 0.314 | 0.736 |
| Saccharomycetes | Fraction | 0.721 | 0.412 |
|  | Grassland type | 0.695 | 0.518 |
|  | Fraction*Grassland type | 1.155 | 0.348 |
| Rhizophlyctidomycetes | Fraction | 4.020 | 0.068 |
|  | Grassland type | 1.617 | 0.239 |
|  | Fraction*Grassland type | 1.435 | 0.276 |
| Ustilaginomycetes | Fraction | 1.497 | 0.245 |
|  | Grassland type | 82.801 | 0.000** |
|  | Fraction*Grassland type | 1.801 | 0.207 |
| Spizellomycetes | Fraction | 0.050 | 0.828 |
|  | Grassland type | 1.641 | 0.234 |
|  | Fraction*Grassland type | 0.655 | 0.537 |
| Exobasidiomycetes | Fraction | 2.853 | 0.117 |
|  | Grassland type | 2.681 | 0.109 |
|  | Fraction*Grassland type | 3.262 | 0.074 |
| Olpidiomycetes | Fraction | 2.251 | 0.159 |
|  | Grassland type | 0.743 | 0.496 |
|  | Fraction*Grassland type | 0.541 | 0.596 |
| Geoglossomycetes | Fraction | 0.909 | 0.359 |
|  | Grassland type | 1.086 | 0.369 |
|  | Fraction*Grassland type | 0.929 | 0.422 |
| Lecanoromycetes | Fraction | 0.127 | 0.728 |
|  | Grassland type | 0.858 | 0.448 |
|  | Fraction*Grassland type | 1.738 | 0.217 |
| Geminibasidiomycetes | Fraction | 1.000 | 0.337 |
|  | Grassland type | 1.000 | 0.397 |
|  | Fraction*Grassland type | 1.000 | 0.397 |

Fraction represents grazing exclusion and grazing; Grassland type represents temperate desert、temperate steppe and mountain meadow.
